# Unsupervised machine-learning method for improving the performance of ambulatory fall-detection systems

**DOI:** 10.1186/1475-925X-11-9

**Published:** 2012-02-16

**Authors:** Mitchell Yuwono, Bruce D Moulton, Steven W Su, Branko G Celler, Hung T Nguyen

**Affiliations:** 1 Faculty of Engineering and IT, University of Technology Sydney, NSW, 2007, Australia; 2 ICT Centre, CSIRO, Marsfield, NSW, 2122, Australia

## Abstract

**Background:**

Falls can cause trauma, disability and death among older people. Ambulatory accelerometer devices are currently capable of detecting falls in a controlled environment. However, research suggests that most current approaches can tend to have insufficient sensitivity and specificity in non-laboratory environments, in part because impacts can be experienced as part of ordinary daily living activities.

**Method:**

We used a waist-worn wireless tri-axial accelerometer combined with digital signal processing, clustering and neural network classifiers. The method includes the application of Discrete Wavelet Transform, Regrouping Particle Swarm Optimization, Gaussian Distribution of Clustered Knowledge and an ensemble of classifiers including a multilayer perceptron and Augmented Radial Basis Function (ARBF) neural networks.

**Results:**

Preliminary testing with 8 healthy individuals in a home environment yields 98.6% sensitivity to falls and 99.6% specificity for routine Activities of Daily Living (ADL) data. Single ARB and MLP classifiers were compared with a combined classifier. The combined classifier offers the greatest sensitivity, with a slight reduction in specificity for routine ADL and an increased specificity for exercise activities. In preliminary tests, the approach achieves 100% sensitivity on in-group falls, 97.65% on out-group falls, 99.33% specificity on routine ADL, and 96.59% specificity on exercise ADL.

**Conclusion:**

The pre-processing and feature-extraction steps appear to simplify the signal while successfully extracting the essential features that are required to characterize a fall. The results suggest this combination of classifiers can perform better than MLP alone. Preliminary testing suggests these methods may be useful for researchers who are attempting to improve the performance of ambulatory fall-detection systems.

## Background

Falls are recognised by the World Health Organization as a major cause of hospitalization of older people [1]. If no preventative measures are undertaken, it is estimated that costs associated with fall-related trauma will double over the next 20 years [1].

Ambulatory accelerometer devices are currently capable of detecting falls in a controlled environment, and these devices are also potentially useful for assessing gait and tremor in older people with Parkinson's disease [2,3]. Research regarding accelerometer-based fall detection typically uses thresholding algorithms [4-6]. Those algorithms typically determine if a person experiences an acceleration above a certain value - that is, an impact acceleration as a person hits the ground - and sometimes combine this with an approach for measuring whether the impact is followed by a period of lying down/not moving. However, research suggests that those approaches can tend to have limited sensitivity for soft-falls (for example, where a person falls against a wall) and break-falls (for example, where a person reduces the impact of their fall by putting out an arm) [e.g. [7]]. Current fall-detection approaches can also tend to result in poor specificity, in part because impacts can be experienced as part of ordinary daily living activities.

The fall-detection work undertaken at the University of Technology Sydney is part of a larger research program focused on health technologies that also focuses on issues associated with sensor-transceivers that stream data such as heart rate, electrocardiogram (ECG), oxygen saturation, body temperature, and body position [8-11].

## Method

The method described here builds on prior work including the work of Shi and others who combined threshold techniques and Support Vector Machines (SVM) to improve the performance of fall detection classifiers [7]. Our approach attempts to improve the specificity even while only using one sensor device, a waist-worn tri-axial accelerometer.

**Figure 1:**
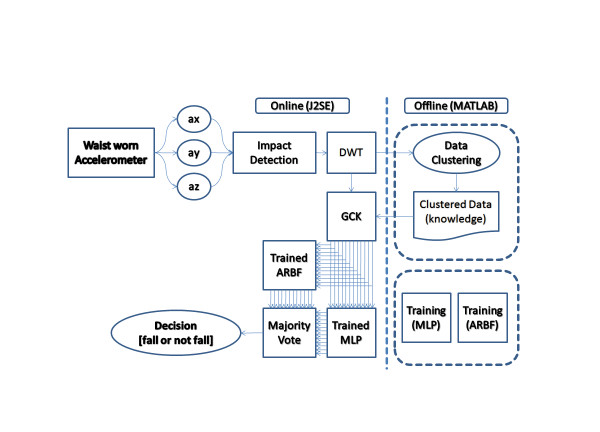
**Block diagram of the system**. The block diagram shows the flow, starting with the accelerometer and ending with a decision as the whether or not a fall has been detected.

A block diagram describing the whole data processing scheme can be seen in Figure 1. Key steps in the process include real time testing of whether the magnitude of acceleration at any given moment is greater than a specified threshold. Each time the threshold is exceeded, an interval of the signal is instantiated and queued. A third order Discrete Wavelet Transform (DWT) transform is applied to each interval in the queue, and features are extracted. The extracted features are passed to the classifiers.

The feature extraction stage makes use a custom method we refer to as Gaussian distribution of Clustered Knowledge (GCK) signal generation. Clustering is done with Regrouping Particle Swarm Optimization (RegPSO). Classification is undertaken by a newly developed "Augmented Radial Basis Function" (ARBF) neural network [12] alongside a multilayer perceptron (MLP).

The input data consists of sampled acceleration values in three dimensions. The main accelerometer module used is the RD-3152 MMA7260Q - Zstar2 from Freescale Semiconductor. The module provides 3-axis acceleration data using an MMA7260Q accelerometer set to ± 6 g sensitivity range. Wireless communication is established using a ZigBee protocol 2.4 GHz band to communicate with the receiver board [13]. The accelerometer sensor is placed inside the right pocket of a vest.

At the receiving end of the wireless link, the samples are handled in real time using Java2SE and Matlab. Each signal has a length of 5 seconds sampled with 20Hz sampling frequency. Signals are divided into 2 classes: fall signals and Activities of Daily Living (ADL) signals.

Data preprocessing is undertaken in three steps: uncommon acceleration detection, normalization, and data filtering.

**Figure 2:**
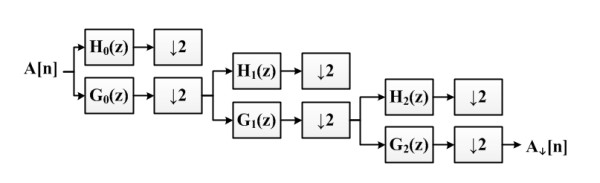
**The application of the Haar Discrete Wavelet Transform**. Convolutions between the original signal A[n] and low pass filters G0, G1, and G2 produces down sampled signal A_↓_[n]. ↓2 block denotes down-sampling, which increase the sample time by two, effectively reducing the number of samples.

• Uncommon acceleration detection: uncommon accelerations, regardless of whether they are due to falls or not, can be observed when the magnitude of acceleration is above a specified threshold. When an uncommon acceleration occurs at time τ, a window is constructed at τ ± 2.5 *s *and acceleration data in that window is pushed to the classification queue.

• Normalization: raw acceleration data $\vec{a}\left(t\right)$ has an offset due to the static force of gravity that differs depending on orientation of the accelerometer. Acceleration signals are normalized by subtracting every sample from $\vec{a}\left(t=0\right)$.

• Data Filtering: the DWT decomposes discrete time signals using a digital filter approach. The DWT computes successive convolutions between input signal with discrete low pass and high pass filters [14]. The application of this filter in the system can be seen in Figure 2. The DWT filters the acceleration signal and down-samples it up to the third order using Haar wavelets. The intention is to reduce the signal complexity while still providing sufficient relevant information to the classifiers.

**Figure 3:**
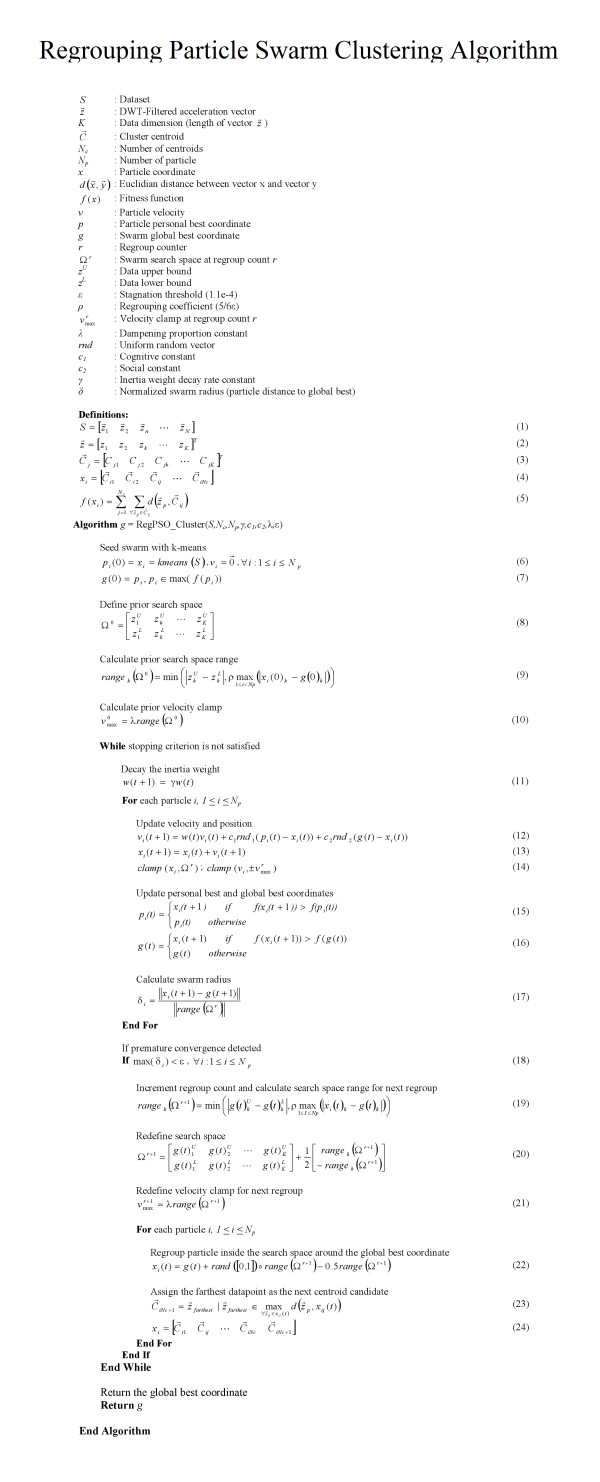
**The clustering algorithm**. A RegPSO method is used to cluster N vectors $\vec{z}$ in the dataset S (1).

Previous work suggests that K-means Particle Swarm Optimization (PSO) can be a reliable tool for data clustering [15]. The approach was originally introduced by Kennedy and Eberhart [16]. It has two base models: Local Best (*lbest*) PSO and Global Best (*gbest*) PSO. Our approach makes use of the *gbest *PSO method. Each particle has *x_i_*: *current coordinate, v_i_*: *current velocity*, and *p_i_*: *personal best coordinate*. The Regrouping Particle Swarm Optimization (RegPSO) approach was proposed by Evers and Ghalia in 2009. RegPSO is designed to remedy premature convergence and stagnation due to local minima problems [17]. We use a RegPSO method to cluster *N *vectors $\vec{z}$ in the dataset *S *(1). The clustering algorithm is given in Figure 3.

The new GCK method was inspired by Monte Carlo approaches. The GCK method is intended to help classify clustered patterns of statistical characteristics. It refers incoming input signals to cluster centroids, and multiplexes them based on the Gaussian characteristics of the clusters. Each input signal $\bar{\gamma }$ is queried against the cluster centroids and passed through a Radial Basis Kernel (25) to get the rate of membership $\theta_{i}\left(\bar{\gamma }\right)$. The cluster with the highest rate of membership, Cluster *I*, is selected as the GCK seed. A knowledge signal  is obtained by generating a vector of Gaussian random numbers with mean ${\bar{\mu }}_{I}$ and standard deviation ${\bar{\sigma }}_{I}$ (26). The generated GCK signal  is fused with $\bar{\gamma }$ using a significance ratio of *A*:*B *(typically 0.8:0.2) to create signal $\bar{\vartheta }\left(\bar{\gamma }\right)$ (27).

(25) $$\theta_{i}\left(\bar{\gamma }\right)=e^{\frac{∥\bar{\gamma }-\mu_{i}∥}{2{\sigma_{i}}^{2}}}$$

(26) $$ȳ\left(\bar{\gamma }\right)=N\left({\bar{\mu }}_{I},{\bar{\sigma }}_{I}\right),I\in \text{max}\theta_{i}\left(\bar{\gamma }\right)|1\leq i\leq N_{c}$$

(27) $$\bar{\vartheta }\left(\bar{\gamma }\right)=\frac{A\bar{\gamma }+Bȳ\left(\bar{\gamma }\right)}{A+B}$$

**Figure 4:**
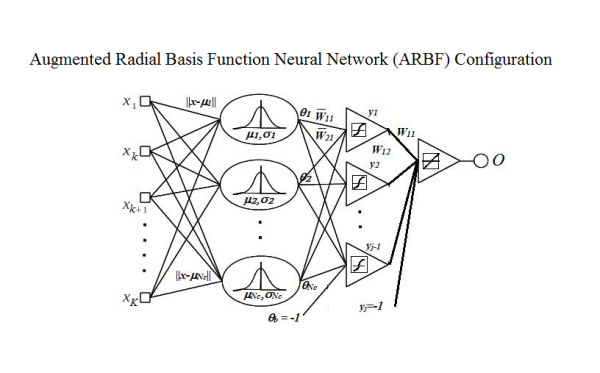
**The Augmented Radial Basis Function classifier**. The ARBF classifier consists of an RBF layer and an MLP augmentation layer.

Augmented Radial Basis Function neural networks (ARBF) have previously been used in time signal classification of head movement patterns, with promising results [12]. ARBF consists of an RBF layer and an MLP augmentation layer, shown in Figure 4. ARBF is reported to have a sensitivity advantage over conventional RBF and a specificity advantage over MLP [12].

The ARBF function uses a Gaussian radial basis kernel. It can be described as a K-dimensional Gaussian distribution, where K is the number of dimensions of the input. The output of the RBF layer is a vector where μ_*n *_and σ_*n *_correspond to cluster centroids and the standard deviation of each RBF node. The RBF centroids are optimized using RegPSO. The MLP layer uses a sigmoid kernel in the hidden layer and a linear kernel in the output layer. No normalization method is required at this stage because the RBF layer has already normalized the input signals from 0 to 1. The MLP layer is trained with resilient back-propagation. This combination of MLP and ARBF was used because of each classifier's different characteristics. MLP networks tend to perform better in global generalization, while RBF-kernel based classifiers such as ARBF tend to perform better in local generalization [12]. The ensemble receives the collection of signals consisting of the original signal $\bar{\gamma }$ and *N *GCK-Fused signals $\bar{\vartheta }$, and each neural network outputs *N *+ 1 classifications of the input vectors. The outputs are then combined based on majority vote.

A brief summary of the steps taken is as follows:

1. Pre-process the data;

2. Create the clustered-knowledge database using RegPSO;

3. Separate the In-group fall data and ADL data into training and validation sets with ratio of 4:1;

4. Train MLP using resilient back propagation;

5. Train ARBF;

6. Create an RBF layer with cluster centroids taken from the clustered-knowledge result from RegPSO;

7. Pass the pre-processed data to the RBF layer;

8. Pass the output of the RBF layer to MLP layer;

9. Train the MLP with resilient back propagation;

10. Merge the RBF layer with the MLP layer.

Table 1 provides a description of each set of data. The project was conducted in compliance with the Helsinki Declaration, and in accordance with the University of Technology Sydney (UTS) research guidelines and clearance granted by the UTS Human Research Ethics Committee.

**Table 1:** The data

Data	Participants	Signals
In-group fall data	Collected from 5 healthy volunteers, 2 females and 3 males. Volunteers aged between 19 and 28 years.	293 fall signals were collected. Of these, 153 signals were used for training, and 140 signals used for testing (in-group performance)

Out-group fall data	Collected from 3 different healthy male volunteers whose data was not included in the training data. Volunteers aged between 19 and 28 years.	This set included 85 signals, all used to test "out-group" performance. The term "out-group" is used to indicated that these people's data was not used as training data.

The Activities of Daily Living (ADL) training data	Collected from 3 people. A total of 8 hours of ADL data was collected in a home environment. An additional hour of exercise data was recorded from 2 people in a gym environment. Volunteers aged between 19 and 28 years.	1831 ADL signals were collected. 1000 randomly selected ADL signals were used for the training set while 831 were used for testing. Of the 1000 randomly selected signals used for training, 750 related to ADL routine, and 250 related to ADL exercise. Of the 831 signals used for testing, 400 related to ADL routine and 381 related to ADL gym exercise.

Validation set		Taken randomly from the training set with the ratio of training versus validation = 4:1.

## Results and Discussion

**Figure 5:**
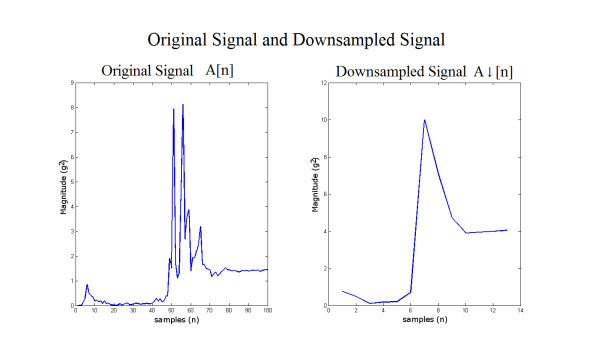
**Original signal and down-sampled signal**. Signal in the left is original signal, signal in the right is downsampled signal. The dimension has been reduced from 100 to 13 while the important features are conserved.

**Figure 6:**
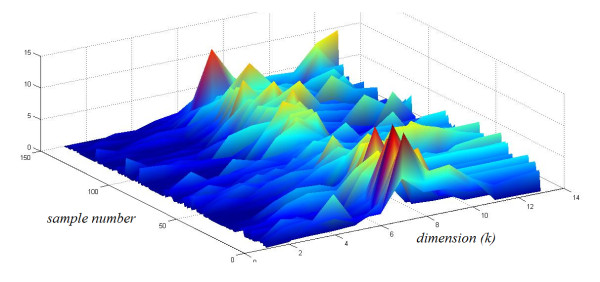
**Clustered pre-processed fall signals**. In this figure 150 signals are stacked for visualization purpose. The sample number indicates sample number 1 to 150. After third order DWT, the original signal dimension is reduced to 13 dimensions.

**Figure 7:**
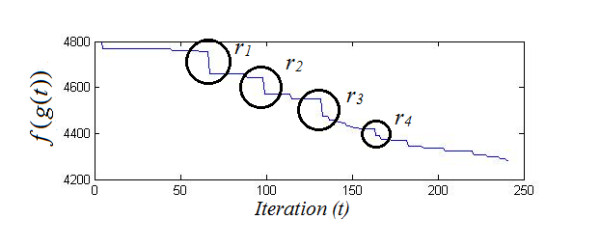
**The progression through 250 iterations of the classification algorithm**. g(t) is the best cluster combination at time t, f(g(t)) is the fitness function, r_n _denotes regroup episodes. Note that f(g(t)) improves greatly at each regroup episode.

**Figure 8:**
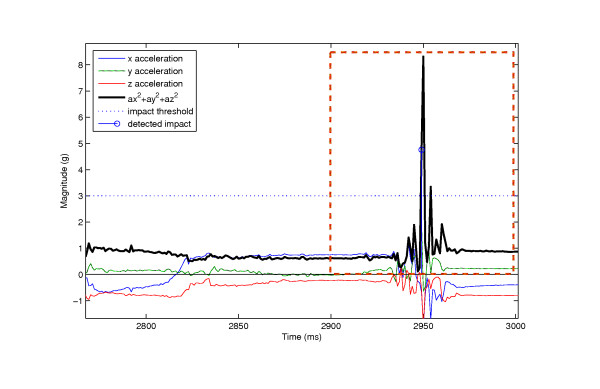
**Example of a fall signal**. A fall signal is characterized by the high impact magnitude and posture change, determined by the drift of the starting acceleration and the final acceleration.

**Figure 9:**
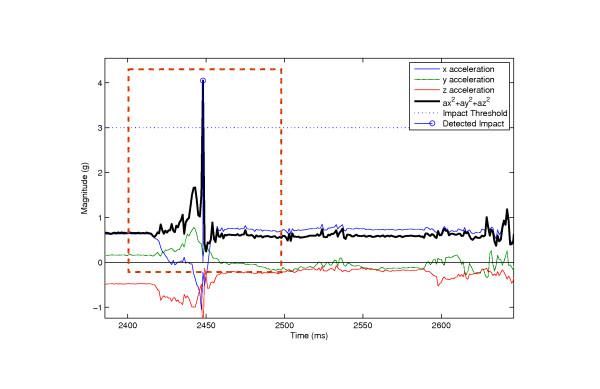
**Example of ADL Sitting Down signal**. Sitting down signal is characterized by an impact and no posture change, determined by the indiscriminate drift of the starting acceleration and the final acceleration.

An example of the output from the Haar DWT third order filtering processing stage is given in Figure 5 The original signal A[n] is shown on the left, and the processed signal A_↓_[n] is shown on the right. It can be seen that the processed signal appears to have reduced complexity, but still retains the essential features. Figure 6 shows 150 pre-processed fall signals stacked together. Figure 7 provides an example of the progression though the first 250 iterations of the classification algorithm. Figure 8 shows a fall signal, and Figure 9 shows an ADL sit down signal.

**Figure 10:**
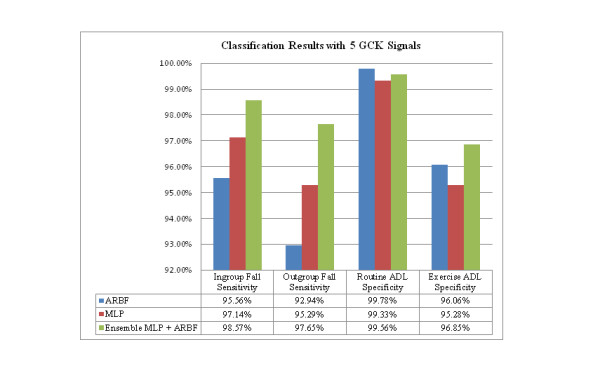
**Results - comparison of classifiers**. Ensemble MLP + ARBF generally perform better than the individual MLP or individual ARBF classifier.

**Figure 11:**
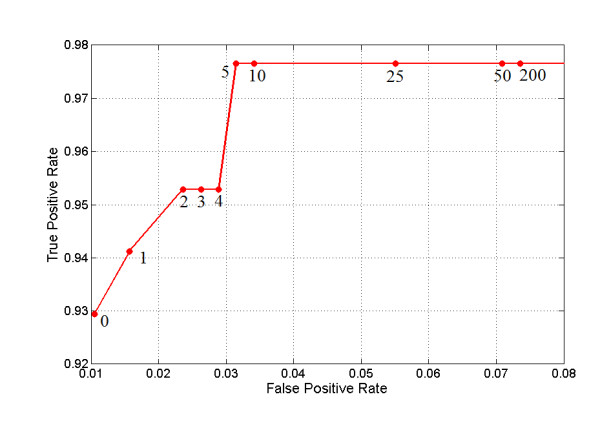
**The effects of GCK on classifier performance**. True Positive Rate is measured using Out-group fall data, the false positive rate is measured using exercise data. Using more than 5 GCK does not seem to improve sensitivity. A reduction in specificity can be observed as the number of GCK signals is increased.

Table 2 shows the results comparing ARB + MLP combined with ARB alone and MLP alone, where the number of GCK signals equals 5. The table shows that the combined classifier offers the greatest sensitivity, with a slight reduction in specificity for routine ADL and an increased specificity for exercise activities. Figure 10 shows the sensitivity and specificity of each approach where GCK = 5. This number was selected because trials indicated that sensitivity improves up to GCK = 5 but stays the same at greater than 5, while specificity of the system decreases when GCK greater than 5 is used. The GCK effects to the classifier performance can be seen in Figure 11.

**Table 2:** Comparison of the results for the three classifier method

Classifier Scheme	Ingroup Fall Sensitivity (N = 140)	Outgroup Fall Sensitivity (N = 85)	Routine ADL Specificity (N = 450)	Exercise ADL Specificity (N = 381)
ARBF	95.56%	92.94%	99.78%	96.06%

MLP	97.14%	95.29%	99.33%	95.28%

Ensemble MLP + ARBF	98.57%	97.65%	99.56%	96.85%

In preliminary tests, the approach achieves 100% sensitivity on in-group falls, 97.65% on out-group falls, 99.33% specificity on routine ADL, and 96.59% specificity on exercise ADL.

Limitations applicable to these results include the following. First, the number of subjects is relatively small. Second, the method for acquiring the falls data did not include truly accidental falls - the falls data was acquired when subjects deliberately fell onto a mattress. Third, the ADL data included only activities around the home and activities in the gym - data relating to ordinary work, transportation and other non-home activities would likely be more representative of some people's typical daily activities. Fourth, the data was acquired from people aged 19-28 years - it would be preferable for future work to include people from older age brackets.

These results are an exploratory step towards gaining knowledge about potential elements of a fall detection system. The implications of the results are somewhat limited due to limitations of the data acquisition processes. Notwithstanding, the results suggest the methods described here warrant further development and experimental investigation.

A further implication of these results is that some of the methods described here may also be applicable for body movement analysis and gait analysis relating to conditions that affect balance such as Parkinson's disease.

Future research will include acquiring data from different age groups, and developing methods to make use of data from ambulatory devices that include gyroscopes and a magnetometer.

## Conclusions

Preliminary testing suggests the methods described here are noteworthy particularly for researchers who are attempting to improve the performance of ambulatory fall-detection systems. The methods should also be of interest for researchers who use (or are considering using) accelerometers to measure body movement. The pre-processing and feature-extraction steps appear to simplify the signal while successfully extracting the essential features that are required to characterize a fall. The results suggest that the approach used here performs better than MLP alone.

## Competing interests

The authors declare that they have no competing interests.

## Authors' contributions

MY carried out the research. MY and BM drafted the manuscript. BM, SS, BC and HN supervise the research. All authors read and approved the final manuscript.
